# Influence of Chemical Composition on Structure and Mechanical Properties of Vacuum-Carburized Low-Alloy Steels

**DOI:** 10.3390/ma17020515

**Published:** 2024-01-21

**Authors:** Paweł Kochmański, Renata Chylińska, Paweł Figiel, Sebastian Fryska, Agnieszka E. Kochmańska, Magdalena Kwiatkowska, Konrad Kwiatkowski, Agata Niemczyk, Justyna Słowik, Wojciech Maziarz, Łukasz Rogal, Konrad Dybowski, Jolanta Baranowska

**Affiliations:** 1 Faculty of Mechanical Engineering and Mechatronics, West Pomeranian University of Technology in Szczecin, Av. Piastów 19, 70-310 Szczecin, Polandkonrad.kwiatkowski@zut.edu.pl (K.K.); jolanta.baranowska@zut.edu.pl (J.B.); 2 Institute of Metallurgy and Materials Science, Polish Academy of Sciences, Reymonta 25 Str., 30-059 Krakow, Poland; maziarz.w@imim.pl (W.M.); rogal.l@imim.pl (Ł.R.); 3 Institute of Materials Science and Engineering, Lodz University of Technology, 1/15 Stefanowskiego St., 90-537 Lodz, Poland

**Keywords:** low-pressure carburizing, gas carburizing, low-alloy structural steel, fatigue strength

## Abstract

This study presents research results concerning the vacuum carburizing of four steel grades, specifically conforming to European standards 1.7243, 1.6587, 1.5920, and 1.3532. The experimental specimens exhibited variations primarily in nickel content, ranging from 0 to approximately 3.8 wt. %. As a comparative reference, gas carburizing was also conducted on the 1.3532 grade, which had the highest nickel content. Comprehensive structural analysis was carried out on the resultant carburized layers using a variety of techniques, such as optical and electron scanning, transmission microscopy, and X-ray diffraction. Additionally, mechanical properties such as hardness and fatigue strength were assessed. Fatigue strength evaluation was performed on un-notched samples having a circular cross-section with a diameter of 12 mm. Testing was executed via a three-point bending setup subjected to sinusoidally varying stresses ranging from 0 to maximum stress levels. The carburized layers produced had effective thicknesses from approximately 0.8 to 1.4 mm, surface hardness levels in the range of 600 to 700 HV, and estimated retained austenite contents from 10 to 20 vol%. The observed fatigue strength values for the layers varied within the range from 1000 to 1350 MPa. It was found that changing the processing method from gas carburizing, which induced internal oxidation phenomena, to vacuum carburizing improved the fatigue properties to a greater extent than increasing the nickel content of the steel.

## 1. Introduction

Presently, many key machine components, such as shafts, large roller bearings, and parts of tools and gears, are manufactured from case-hardened alloy steels. Gears serve a broad spectrum of applications. For instance, high-power wind turbines [1] typically incorporate a gearbox that converts the low-speed rotation of the rotor shaft into a higher rotational speed transmitting high torque. Diverse alloy formulations, heat treatments, and thermo-chemical processes have been devised to achieve the required combination of properties. Generally, gears undergo a case-carburizing treatment to improve their performance. The heat treatment procedure associated with case carburizing is intricate, demanding a high level of technical expertise and an in-depth comprehension of material properties [2].

One of the prevailing carburizing techniques is still employed; e.g., within the European automotive sector, it involves gas carburizing conducted in endothermic atmospheres or generator-free atmospheres directly produced within the furnace retort. The furnace atmosphere is a mixture of gases containing carburizing and reducing components, such as carbon monoxide and hydrogen, alongside oxidizing components, such as carbon dioxide and water. The presence of oxidizing components promotes the occurrence of two phenomena, internal oxidation (IGO/IGA—Intergranular Oxidation/Intergranular Attack) and decarburization that significantly and adversely impact fatigue strength.

The potential for internal oxidation is particularly notable in steels alloyed with elements such as chromium, manganese, and silicon, which possess a high affinity for oxygen. These elements are intentionally incorporated into steel for products subjected to substantial mechanical loads and are aimed at augmenting the core hardenability, thereby bolstering the strength. Internal oxidation engenders the formation of a zone containing High-Temperature Transformation Products (HTTP), leading to a decline in hardness and, consequently, a reduction in fatigue strength, as documented in several studies [3,4,5]. The extent of internal oxidation and decarburization increases proportionally with the depth of the carburized layer, which ranges from 25 to 75 µm [3]. Numerous research reports have indicated a potential reduction in fatigue strength by up to 50% due to these effects [3]. Despite the notable drawbacks of gas carburizing concerning service properties, it is still widely used in industries due to its process efficiency, which surpasses that of low-pressure methods executed at similar temperatures.

A technological solution without the main disadvantages of gas processes is offered by vacuum carburizing [6,7,8], encompassing Low-Pressure Carburizing (LPC) and Low-Pressure Carbonitriding (LPCN). These vacuum techniques employ carbon (and nitrogen) carriers devoid of oxygen, such as the hydrocarbons acetylene, ethylene, methane, and propane. In vacuum carbonitriding, ammonia typically serves as the nitrogen carrier. This methodology facilitates a shorter process duration through elevated temperatures up to 1100 °C. Ammonia addition during the heating phase within the temperature range of 400–700 °C restricts the growth of austenite grains by releasing nitrides, thereby inhibiting austenite grain coarsening [9]. Moreover, as suggested by the literature reports [4,10,11], the presence of nitrogen in diffusion layers improves ductility, potentially benefiting fatigue strength under dynamic loads. The presence of nitrogen alters the start and finish temperatures of martensitic transformation, increasing the proportion of retained austenite after hardening.

The impact of the presence of retained austenite and its proportion on the mechanical properties of carburized and carbonitrided layers remains contentious, with the literature presenting conflicting information. Retained austenite, being considerably softer than martensite, may be disadvantageous in components operating under conditions where friction occurs. Divergence in the literature arises in appraising its effect on fatigue strength under distinct mechanical load scenarios. Certain researchers contend that retained austenite has considerable advantages as, under external stress conditions from plastic deformation, it transforms into martensite that exhibits greater ductility compared to thermally formed martensite [12]. Furthermore, retained austenite hinders crack propagation [13], which suggests that the fatigue strength increases under high contact stresses [14]. Conversely, other researchers postulate that martensite induced by deformation in carburized layers causes brittleness, thereby deeming it unfavorable [15]. References [16] and [17] posit a significant reduction in fatigue strength that occurs due to the presence of retained austenite within the layer.

The chemical composition of alloy steels used for carburizing can vary based on the specific application and desired properties [2]. Some common alloying elements found in carburizing steels include manganese, chromium, nickel, and molybdenum. These alloying elements are strategically combined to enhance the steel’s hardenability, wear resistance, toughness, and core strength. The exact composition and proportions of these alloying elements can vary based on the specific grade of steel and its intended application, with manufacturers tailoring the composition to achieve the desired balance of properties for the end-use requirements of the carburized steel. Chromium contributes to the hardenability of steel, enabling it to achieve a deeper and more uniform hardness upon carburizing. Molybdenum enhances the hardenability and strength of steel. It helps in achieving a finer grain structure during heat treatment, thereby improving the mechanical properties of the carburized steel, such as strength, toughness, and resistance to fatigue and fracture. Nickel is known for its ability to improve toughness, strength, and ductility in steel. In carburizing steels, nickel aids in enhancing the core strength, toughness, and impact resistance, which are essential for components subjected to high stress and impact loads. However, nickel is a costly element whose price can vary, leading to the exploration of alternative steel compositions possessing lower nickel content [18].

This article concerns an investigation of four grades of low-alloy carburizing steel with varying nickel content, focusing on their response to vacuum and gas carburizing. The aim of this study is to establish correlations between composition, microstructure, and mechanical properties, in particular, fatigue properties.

## 2. Materials and Methods

Four grades of low-carbon alloy steels for carburizing, with chemical compositions given in Table 1, were used for this experiment. These grades are commonly used to produce heavy-duty machine elements such as shafts, gears, large-sized bearings, etc., which are applied in various industries, such as energy, automotive, and mining. All steel grades used contained chromium in the amounts from 1 to approximately 2 wt. %, and the nickel concentration varied from 0.1 to 3.8 wt. %. Three steel grades contained molybdenum in amounts above 0.2 wt. %.

**Figure 1:**
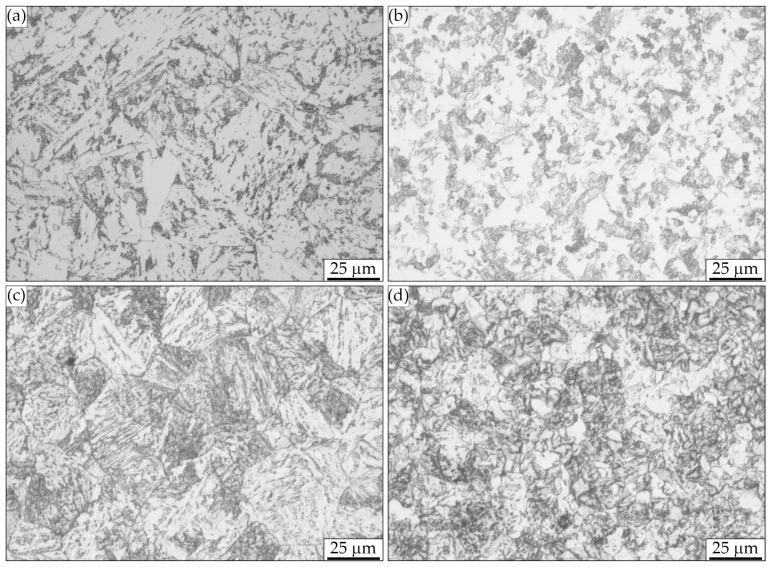
Microstructures of steels used in this experiment in as-received condition. Light optical microscopy. (**a**) 1.3532. (**b**) 1.5920. (**c**) 1.6587. (**d**) 1.7243.

Both the microstructures (Figure 1) and the hardness (Table 2) of the steel as-received differed significantly. Steel 1.5920 had the lowest hardness (158HV), resulting from a full or isothermally annealed microstructure (Figure 1b). The highest hardness (286HV) was found for steel 1.7243, which had a very fragmented structure, most likely during thermo-mechanical processing (Figure 1d). The hardness of the remaining grades, i.e., 1.3532 and 1.6587, was approximately 250 HV, and their microstructure, although slightly different, was acicular in nature: ferritic–bainitic type (Figure 1a) and upper bainite type (Figure 1c), respectively. The grain size number of the steel as delivered ranged from 7 to 8 (Table 2).

### 2.1. Low-Pressure Heat Treatment

This study made use of two low-pressure diffusion procedures: LPC (Low-Pressure Carburizing) and LPCN (Low-Pressure Carbonitriding), each followed by double-gas quenching and subsequent low-temperature tempering.

Metallographic and fatigue samples were introduced into a single-chamber vacuum furnace, class LPC+HPGQ (Low-Pressure Carburizing + High-Pressure Gas Quenching, VPT-4022/24IQN, Seco/Warwick, Świebodzin, Poland) for the carburization and quenching processes. The furnace’s working chamber evacuated to a pressure of 10 Pa, was initially heated to 960 °C. In the LPCN process, ammonia was introduced at 400 °C, followed by maintaining an isothermal stop at 700 °C for approximately one hour to facilitate nitriding. The primary objective of the nitriding stage in this process was to mitigate the coarsening of austenite grains and, concurrently, to facilitate the incorporation of nitrogen into the diffusion layer [19,20]. Subsequently, the temperature was raised back to 960 °C, and a combination of process gases—ethylene, acetylene, and hydrogen in volume proportions of 2:2:1—was introduced. The LPC and LPCN processes involved alternating boost (gas mixture dosing) and diffusion (vacuum) stages.

**Figure 2:**
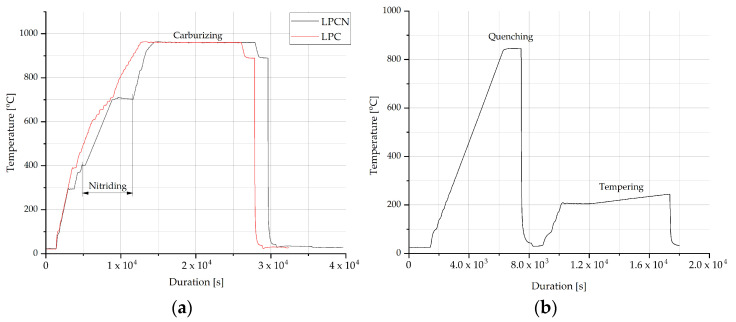
Temperature alternations during (**a**) LPC – red line and LPCN – black line diffusion stage and the first quenching and (**b**) the second quenching and tempering.

Subsequent to the final diffusion stage for both LPC and LPCN, a cooling phase under vacuum (10 Pa) to approximately 890 °C was carried out, followed by isothermal annealing for about 30 min at this temperature. This was followed by high-pressure gas quenching (1.2 MPa) using nitrogen. Subsequently, a second quenching phase was conducted under vacuum conditions for all samples from both LPC and LPCN processes. Austenitization was carried out at a temperature of 840 °C, employing HPGQ with nitrogen. The final step in the heat treatment process involved tempering at a temperature of approximately 200 °C for a duration of 2 h in a vacuum. The recorded temperature variations throughout the heat treatment processes are graphically depicted in Figure 2.

### 2.2. Gas Atmosphere Heat Treatment

**Figure 3:**
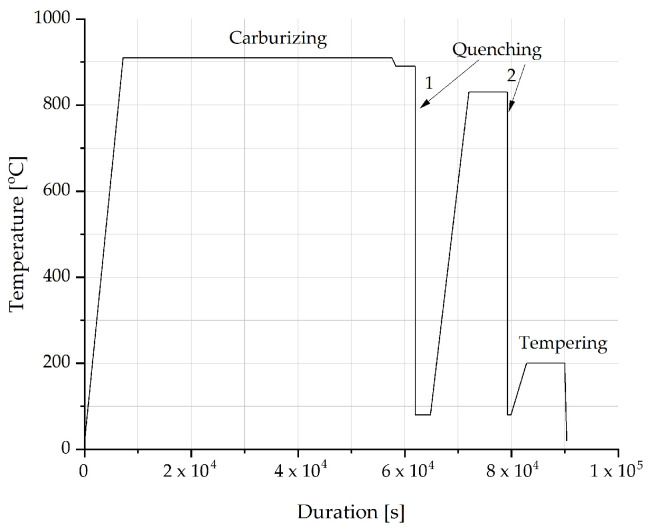
Temperature alternations during gas heat treatment.

As a reference for fatigue testing, samples composed of steel grade 1.3532 (containing the highest nickel content) underwent treatment in a gas endothermic atmosphere. This atmosphere comprised a gas mixture of CO, CO_2_, H_2_, H_2_O, and CH_4_, maintaining a carbon potential within the range from 0.7 to 1.4% C. The gas heat treatment procedure was conducted in a PEKAT-3 furnace (Elterma, Świebodzin, Poland) at a temperature of 916 °C, followed by a double oil quenching process at 80 °C. The final stage involved tempering at approximately 200 °C for a duration of 2 h. Figure 3 illustrates the temperature profile during the gas heat treatment process.

### 2.3. Structural Characterization Protocol

Samples for microstructural examination, after mechanical cutting, were mounted in a conductive resin (Polyfast, Struers, Ballerup, Denmark) and mechanically ground, followed by polishing, using 9, 3, 1, and 0.25 µm diamond suspensions. The microstructures on the cross-sections of the layers were chemically etched using Nital etchant containing 1 cm^3^ of nitric acid and 100 cm^3^ methanol.

Light optical microscopy (LOM) examination was performed using a Nikon Ephiphot 200 (Nikon Corporation., Tokyo, Japan).

The layers were also examined using an FE-SEM SU-70 (Field Emission Scanning Electron Microscopy) microscope (Hitachi, Naka, Japan) and WDS (wavelength dispersive spectrometry)/EDS (energy dispersive spectrometry) X-ray microanalysis using the NORAN™ System 7 from Thermo Fisher Scientific (Madison, WI, USA). The latter consisted of Magnaray (WDS) and UltraDry X-ray detector (EDS) integrated components. The WDS analysis was performed at an accelerating voltage of 10 kV and an electron-beam current of approximately 20 nA using a TAP-diffracting crystal for oxygen. Net-count elemental mappings were acquired with a resolution of 1024 by 768 and a pixel size of 0.04 µm. X-ray microanalysis examinations were performed on flat (not etched) sample surfaces.

TEM (Transmission Electron Microscopy) characterization was performed using a Tecnai FEG G2 F20 Super Twin (FEI Company, Hillsboro, OR, USA) operating at 200 kV. Thin foil specimens were prepared with the FIB (Focused Ion Beam) technique using gallium ions (Quanta 3D, FEI Company, Hillsboro, OR, USA). Comprehensive structural analysis was carried out on the carburized layers using multiple techniques, such as optical and electron scanning and transmission microscopy, as well as X-ray diffraction.

The chemical composition was analyzed using glow-discharge optical emission spectrometry (GDOES) with a Profiler GD2 (Horiba Jobin Yvon, Montpellier, France). The profiles of carbon were determined using GDOES on subsequent parallel sections every 0.1 mm to 0.5 mm from the surface and then every c.a. 0.25 mm up to approximately 2.25 mm.

Retained austenite (RA) measurement using X-ray diffraction (XRD) is a technique commonly employed in materials science and metallurgy to analyze the amount of austenite retained in a metallic structure after heat treatment or during service. The quantification of RA involves the measurement of the relative volume contribution of martensite and austenite using X-ray diffraction (XRD) patterns. In this method, the total integrated intensity of all diffraction peaks for each phase is considered proportional to its volume content. The volume fraction of retained austenite in all the samples after carburizing was evaluated using X-ray Diffraction (XRD) in accordance with the ASTM E975-22 standard [21], using the four-peak method. The measurements were performed on a PW3040/60 X’Pert Pro diffractometer (PANalytical, Almelo, The Netherlands) equipped with a Cu Kα lamp with a wavelength of λ = 0.15406 nm. Measurements were carried out with a voltage of 40 kV and an anode current of 35 mA over an angular range 2Θ varying between 35°÷ 95°, with a step of 0.08° and a counting time of 100 s per step, using a monochromator with a proportional detector on the reflected beam. To determine the volume fraction of RA, the integrated intensities of the two diffraction planes were compared: (200) and (220) for f.c.c. (γ-Fe) with (200) and (211) for b.c.c. (α-Fe), respectively. The acquired data were processed using X’Pert HighScore (v.2.2.1) and ProfileFit (V1.0C) software provided by Panalytical (Almelo, the Netherlands).

### 2.4. Mechanical Properties Characterization Protocol

Superficial hardness measurements were performed with a Vickers indenter (LV700AT, Leco, St. Joseph, MI, USA) at a load of 30 kg, and low-force hardness measurements were performed with a Vickers indenter (Leco LM247AT, Leco, St. Joseph, MI, USA) at a load of 0.5 kg.

Mechanical fatigue tests were performed using an ELectroPulse 10,000 (Instron, Norwood, MA, USA) electrodynamic testing machine with a 10 kN range force gauge.

Fatigue testing was conducted using Instron WaveMatrix 2.0 software in a force control system. The applied loading was sinusoidally alternated from zero to the maximum value. The loading frequency was 20 Hz, and no sample heating was observed at this frequency.

**Figure 4:**
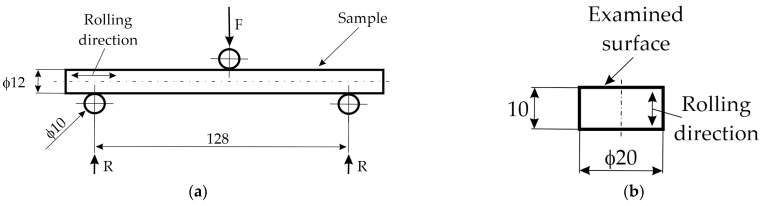
(**a**) Scheme of fatigue testing setup. (**b**) Metallographic sample.

Notchless samples with a circular cross-section and a measuring part with a diameter of 12 mm were bent in a 3-point test (Figure 4a). The supports and forcing pin were rollers with a diameter of 10 mm and a hardness of 62 HRC. The span between the supports was 128 mm. Samples for fatigue strength tests were produced by machining, i.e., turning from bars with a diameter of 20 mm, followed by grinding. The Ra roughness value was approximately 0.2 µm. Samples (Figure 4b) for LOM, SEM, XRD, GDOES, and hardness tests were cut from the same bars from which the fatigue samples were made.

The stress amplitude was selected such that the outer surface layer achieved the assumed maximum stress: tensile in the lower part of the sample and compressive in the upper part. The amplitude coefficient was R = 0, which means that the stress was zero-pulsatory. When the number of cycles exceeded 10^7^ without failure of the sample, the test was stopped. For reference purposes, a full Woehler curve was determined for the gas carburized layer on 1.3532 steel, and on this basis, two stress amplitude values were selected for further tests, i.e., 1350 and 1500 MPa. The bending strength determined for the gas-carburized layer on 1.3532 steel was 2790 MPa, while yield stress (at 0.2%, relative strain) was 2040 MPa. Five samples were tested for each combination of the parameters of steel grade, low-pressure process, and stress value.

## 3. Results

### 3.1. Microstructure

**Figure 5:**
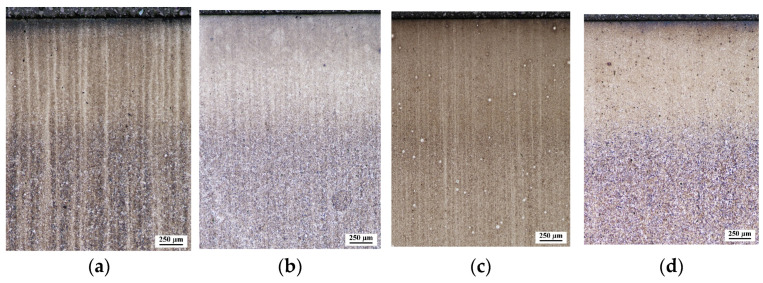

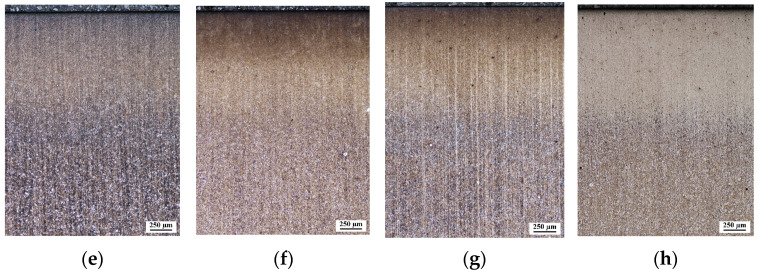
Microstructures of hardened layers from LPC process: (**a**) 1.3532; (**b**) 1.5920; (**c**) 1.6587; (**d**) 1.7243 and LPCN process: (**e**) 1.3532; (**f**) 1.5920; (**g**) 1.6587; (**h**) 1.7243. Light optical microscopy.

**Figure 6:**
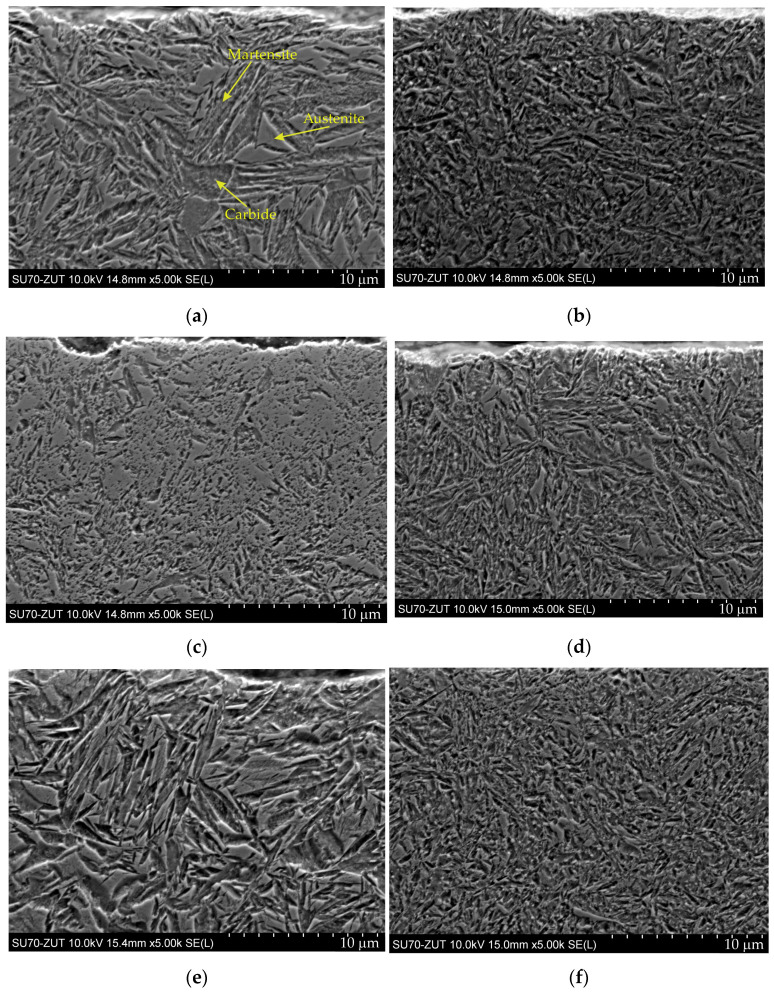

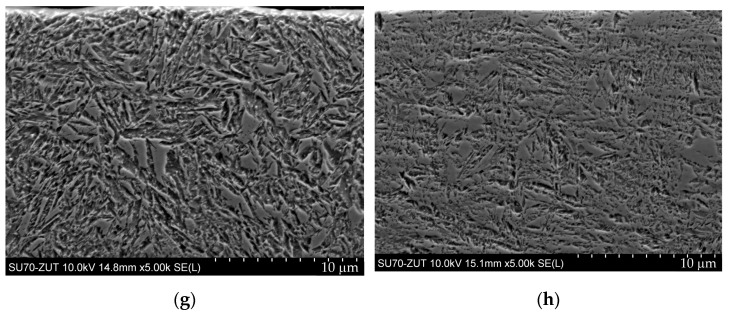
Microstructures of hardened layers from LPC process: (**a**) 1.3532; (**b**) 1.5920; (**c**) 1.6587; (**d**) 1.7243 and LPCN process: (**e**) 1.3532; (**f**) 1.5920; (**g**) 1.6587; (**h**) 1.7243. Secondary electrons, SEM.

The microstructures of the layers produced with the two low-pressure processes are shown in Figure 5 and Figure 6, which show light and scanning electron microscopy, respectively.

Light optical microscopy observations carried out at relatively low magnification (Figure 5) showed banding of the microstructure, especially for steel grades 1.3532 and 1.6587.

For all tested samples, the microstructure of the surface layer at high magnification (Figure 6) was low-tempered fine needle-shaped martensite, containing a certain amount of retained austenite and fine secondary carbides. The microstructure of the core of steel grades 1.3552, 1.5920, and 1.6587 was low-carbon martensite, while in steel 1.7243, the core had a microstructure consisting of bainite and low-carbon martensite. The average grain size number of the prior austenite is shown in Table 3. No secondary cementite network was observed in the surface layer of any tested sample.

**Figure 7:**
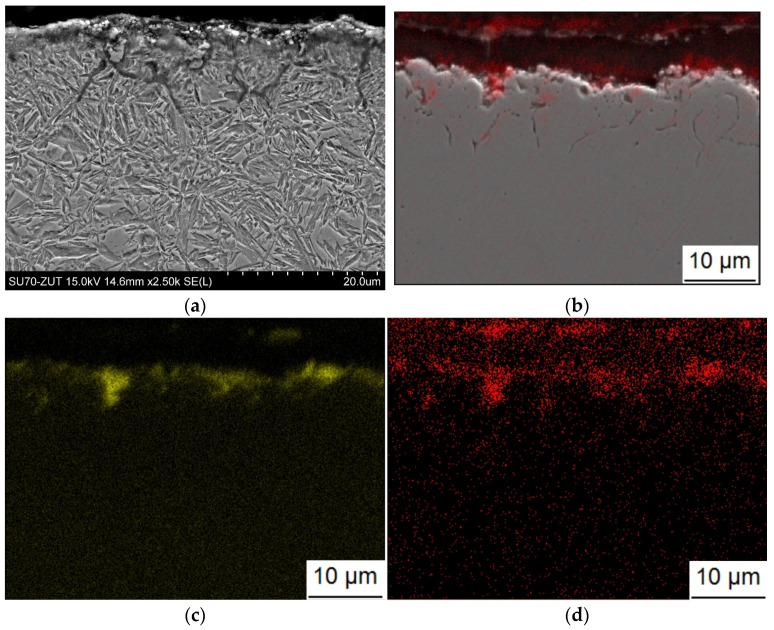
Microstructure of gas heat-treated layer on steel grade 1.3532. (**a**) Secondary electron image, sample etched with Nital. (**b**) Secondary electron image plus oxygen distribution map, WDS. (**c**) Chromium distribution map, EDS. (**d**) oxygen distribution map, WDS.

The microstructure of the layer produced with gas carburizing is shown in Figure 7. The microstructure of this layer consisted of fine-grained martensite with a retained austenite. Near the surface, there was an oxygen-rich zone (Figure 7b,c), with a thickness of several micrometers and a zone of internal oxidation reaching up to approximately 20 micrometers in depth, with an intergranular course along the boundaries of prior austenite grains. An increased concentration of chromium was present in the internally oxidized areas (Figure 7c). No secondary cementite was observed in this layer. Non-metallic inclusions of oxides and a few sulfides were present in the microstructure.

### 3.2. X-ray Diffraction

**Figure 8:**
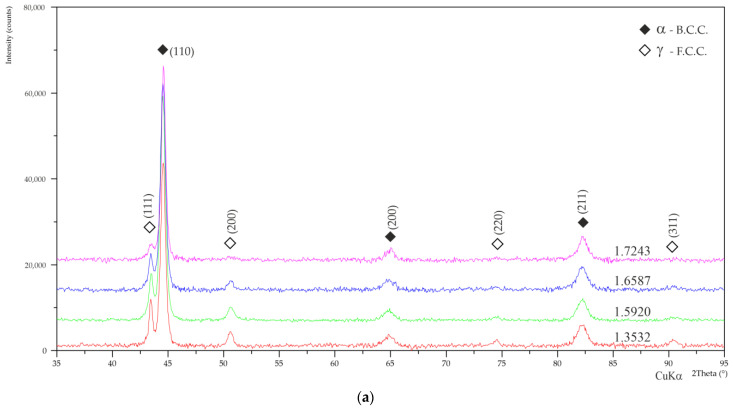

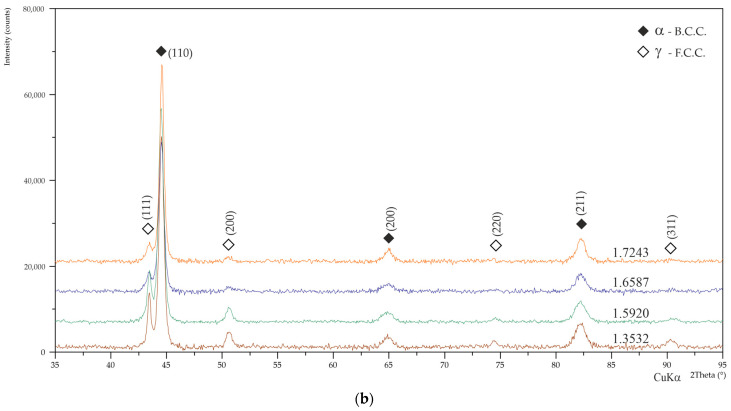
XRD diffraction patterns acquired from layers treated in low-pressure processes: (**a**) LPC; (**b**) LPCN.

Figure 8 presents X-ray diffractograms collected from the surface of layers produced during the low-pressure processes.

In a qualitative sense, all the presented diffractograms were similar to each other, i.e., diffraction peaks coming from the α phase (martensite) with a b.c.c. structure were visible, and the peaks coming from the γ phase retained austenite (f.c.c.). The presence of other phases, such as secondary carbides, was not observed due to their volume fraction being too low. Based on the diffractograms, the retained austenite content was calculated, and the results are shown in Table 4. As expected, the most retained austenite was found in the layers of steel type 1.3532, i.e., with the highest nickel content, and the least was found in the layers of steel 1.7243, which contained the least nickel.

### 3.3. Carbon Profiles

**Figure 9:**
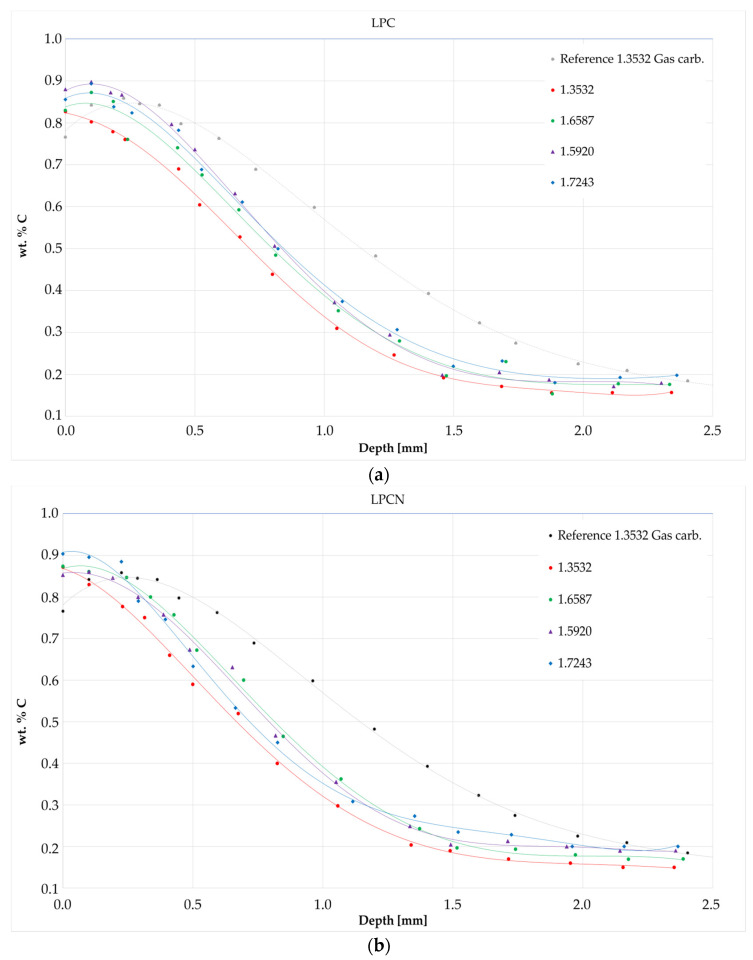
Carbon profiles on layers from heat treatment processes: (**a**) LPC; (**b**) LPCN.

The results of the GDOES analyses are shown in Figure 9. For layers produced by both low-pressure processes, the surface concentration of carbon measured was in the range of 0.85 ± 0.03, while in the gas-carburized layer, it was approximately 0.76 wt. %. A carbon concentration of 0.4 wt. % was measured for all layers from the low-pressure processes at a depth of approximately 0.85 to 1 mm and, in the case of the gas-carburized layer, at a depth of approximately 1.3 mm. Moreover, for the gas-carburized layer, surface decarburization was observable. The layers produced by both low-pressure processes on the richest nickel steel, type 1.3532, have the least amount of carbon.

### 3.4. Hardness Tests

**Figure 10:**
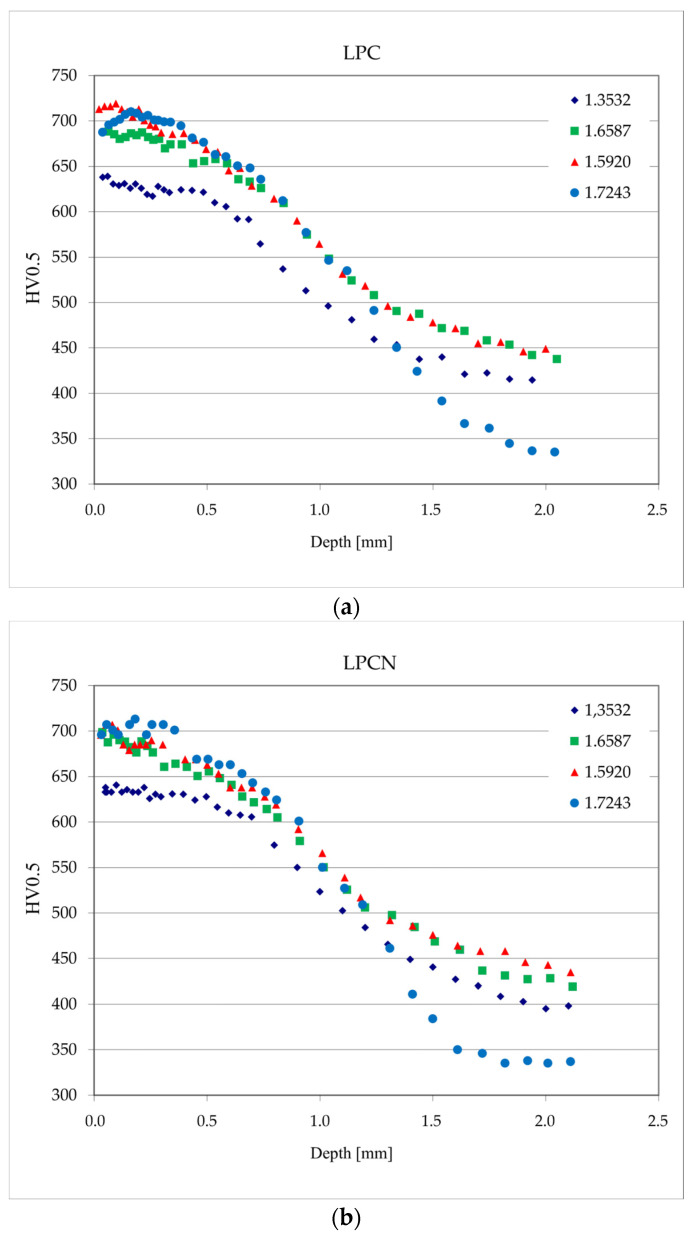
Low-force Vickers hardness profiles for samples treated in low-pressure processes: (**a**) LPC; (**b**) LPCN.

The results of the superficial hardness measurements and hardness profiles in the surface layers obtained using low-force Vickers measurements are shown in Table 5 and Figure 10, respectively. The values presented in Figure 10 are averages from three measurements for each distance from the surface. The uncertainty values did not exceed 5HV for all cases. Considering statistical significance, the values of the superficial hardness of the layers produced in both vacuum processes were similar for the steel grades 1.5920, 1.6587, and 1.17243 and ranged from 676 to 702HV30 (Table 5). However, in the case of layers on type 1.3532 steel, i.e., with the highest nickel content, the surface hardness was significantly lower, i.e., 606 and 622 for LPC and LPCN processes, respectively.

The low-force hardness profiles (Figure 10) for the layers produced on 1.5920 and 1.6587 steels are similar to each other for both low-pressure treatments. A similar profile shape but lower hardness values were observed for both low-pressure treatment layers on the 1.3532 steel. The slope of the hardness profile was the highest for the layers on steel, 1.7243, and the core hardness was the lowest, below 350HV0.5.

CHD (Case Hardness Depth) values, which describe the distance from the surface to the layer with a hardness limit of 550 HV, were determined on the basis of the hardness profiles. The relative case depth was calculated using the formula RCD = CHD/D, where D is the diameter of the sample. The results are presented in Table 6. The CHD value for 1.3532 steel was approximately 0.8 mm, while for other steel grades, it was 1.1 mm.

The CHD value for the layer carburized in the gas process (on the steel grade 1.3532) was 1.4 mm; the relative case depth (RCD) was 0.116, and the superficial hardness was approximately 650HV30.

### 3.5. Fatigue Test

**Figure 11:**
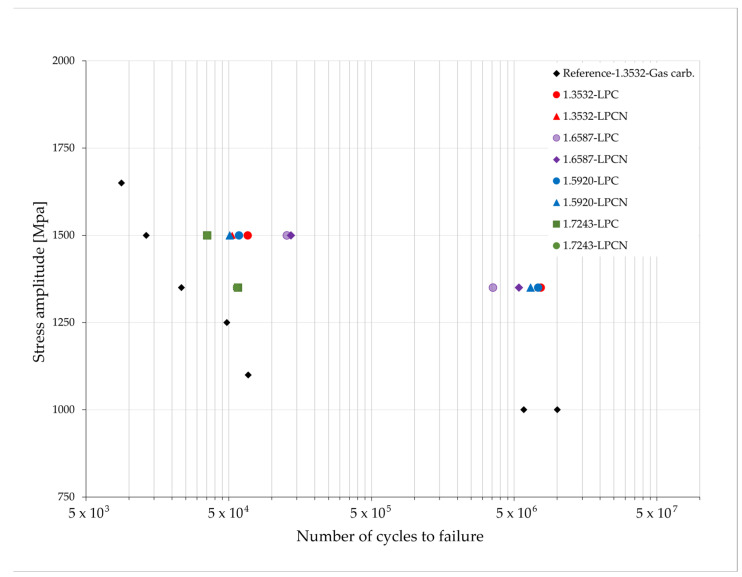
Results of mechanical fatigue tests. S–N graph (Stress—Number of cycles to failure).

The fatigue test results are shown in the form of an S–N (Stress—Number of cycles to failure) graph in Figure 11. The presented cycle number values are the average from five samples. The results obtained for the gas-carburized samples containing IGO indicated that their bending fatigue strength (fatigue limit) was approximately 1000 MPa.

For the samples treated by the low-pressure LPC and LPCN processes, the results obtained for the samples of steel 1.7243 differed significantly from those obtained for the other tested grades, with the number of cycles to fracture being much lower for the 1.7243 steel. The bending fatigue strength limit for the other steel grades is approximately 1350 MPa for both the LPC and LPCN processes.

### 3.6. Transmission Electron Microscopy

**Figure 12:**
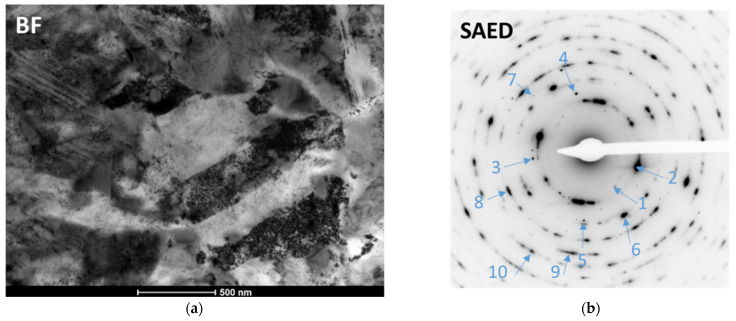
(**a**) TEM bright field microstructure. (**b**) Selected area diffraction pattern acquired from area of BF image (**a**). Ring numbers are marked in blue.

Figure 12 shows the microstructure of a 1.5920 steel sample heat-treated in the LPCN process after the fatigue test. This sample withstood 10M cycles without failure at cyclic stresses of amplitude 1350MPa. The thin foil for TEM testing was taken from the central area of the fatigue strength sample, i.e., from the area where the highest stress occurred during the test. The distance from the surface of the area observed by TEM was approximately 10 micrometers.

The sample had a homogeneous martensite structure in the form of fine needles. A high density of dislocations in the martensite and spherical precipitation of M_3_C-type carbide with a size of approximately 100 nm were present in the microstructure. The strong refining of the microstructure as a result of cyclic deformation during fatigue tests is evidenced by the microstructure shown in Figure 12. The utilization of a diffraction aperture encompassing the complete microstructure area revealed an electron diffraction pattern with an almost annular shape, indicating fragmentation of the microstructure of the examined sample. The calculated interplanar distances (Table 7) based on ring diameter measurements indicated that the sample contained three phases: α-Fe (b.c.c.) in the form of martensite; γ-Fe (f.c.c.) in the form of retained austenite; and, additionally, a ω-type carbide phase in the microstructure of the layer, observed by the authors of publication [23]. The austenite rings were the weakest (often only single reflections were visible), so it can be assumed that this phase was present in the least amount and that the most dominant was the two-phase fragmented microstructure of lath martensite with very fine carbides and a high dislocation density.

## 4. Discussion

When evaluating the fatigue characteristics of case-hardened samples, it is advisable to maintain consistency across multiple factors to ensure a reliable comparison when modifying a single parameter. This is because it is widely acknowledged that several variables impact the fatigue behavior of carburized steels to varying extents. The key variables include the method of the heat treatment and its technological parameters, the steel composition, hardness distribution, carbon concentration profile, case hardening depth, distribution of residual stress, volume of retained austenite, variations in prior austenitic grain size, extent of internal oxidation, surface finishing, specimen shape, and testing method.

In this study, we explored various variables, including the heat treatment methods (specifically, LPC, LPCN, and gas carburizing) and the utilization of four distinct steel grades with differing chemical compositions. Each steel grade investigated contained chromium levels ranging from 1 to approximately 2 wt. % and exhibited varying nickel concentrations, spanning from 0.1 to 3.8 wt. % percent. Additionally, among the examined steel grades, three demonstrated molybdenum content surpassing 0.2 wt. %.

According to the existing literature [3,24,25], it has been advised to maintain the relative case depth within the scope of 0.06 to 0.15, with a strict limit not to surpass 0.20. The increase in the fatigue strength of carburized steel predominantly originates from the development of a high-strength layer and the induction of compressive residual stresses on the surface. The values of the relative casing depth of the samples tested in this experiment ranged from 0.067 to 1.116, so they were within the recommended range.

Regarding the chemical composition, especially the influence of nickel, the GDOES analyses revealed that steel type 1.3532 exhibited comparatively lower carbon saturation in both low-pressure heat treatment procedures when compared to the other examined grades (i.e., 1.5920, 1.6587, 1.7243). The increased nickel content in steel 1.3532 led to decreased surface carbon concentration and a reduced depth at which the carbon concentration reached 0.4 wt. %, resulting in notably decreased surface hardness and CHD (Case Hardened Depth). In low-pressure carburizing processes, nickel increases the carbon activity in austenite, which limits the number of cementite precipitates in the saturation stage of the process, which constitutes an additional source of carbon in the diffusion phase. Therefore, steel 1.3532 is carburized to the smallest extent. Chromium and, to a lesser extent, molybdenum and manganese have the opposite effect on the carbon concentration profile after carburizing. Their presence results in a reduction in carbon activity in austenite, which increases the ability to form precipitates during the saturation phase [9,26].

The authors of this paper [2] indicate a very beneficial effect of the addition of molybdenum on improving the functional properties of carburized steels compared to highly alloyed steels with nickel. However, in our research, such an effect could not be observed in fatigue strength, mainly due to the large dispersion of fatigue test results for steel grades containing nickel.

As anticipated, the chemical composition exerted an influence on the retained austenite content following heat treatment. Specifically, the layers on steel type 1.3532 exhibited the highest level of retained austenite, i.e., over 20 vol. %, due to its elevated nickel (austenite-stabilizing element) content, while the layers on steel 1.7243, possessing the lowest nickel content, displayed the least amount of retained austenite (approximately 10 vol. %).

The combination of a greater volume of retained austenite and reduced carbon concentration may have contributed to the decreased hardness observed in the layer of steel grade of 1.3532.

Another plausible explanation for the decreased surface hardness in layers from the LPC and LPCN processes on 1.3532 steel could be the larger grain size of the martensite and retained austenite in the layers compared to those layers produced on the other tested steel grades.

The following mathematical model [5] was devised specifically for gas carburization of steel devoid of chromium and nickel supplements but incorporating solely molybdenum in AISI8620 (1.5423):

σf = 590 + 36,400 (CHD/D)^1.92^

Using this model gives an anticipated fatigue strength (σf) of 1178 MPa to our gas-treated specimens. However, our experimental determination yielded a value of 1000 MPa for 10^7^ cycles. Even though the model assumes a cycle count of 10^6^, this observation suggests the potential validity of the model even for carburized steel grades that exhibit varying chemical compositions distinct from AISI8620.

The next parameter influenced by the steel’s chemical composition is the core hardness subsequent to heat treatment. For nickel-free steel, the core hardness measured approximately 340HV0.5. In contrast, steel grades containing nickel exhibited a range of core hardness values, from about 400HV0.5 for grade 1.3532 to approximately 440HV0.5 for grades 1.5920 and 1.6587. The core hardness plays a substantial role in determining the fatigue strength of carburized steels. According to the literature [4,5], the optimum core hardness in terms of fatigue strength should be approximately in the range from 300 to 350 HV, and its microstructure should not contain ferrite but only bainite and low-carbon martensite. The fatigue strength test results presented in this article did not confirm this, and samples with a significantly higher core hardness, varying in the range from 400 to 440HV, were much more durable in the fatigue test than samples made of steel grade 1.7243.

Although the presented values for the number of cycles, averaged from five measurements, for low-pressure processed samples are below 10^7^, at least two samples within each sample group did not exhibit cracking at this cycle threshold. Not presented in this article, post-test fractographic examinations using the SEM method on prematurely cracked samples revealed the presence of relatively large non-metallic inclusions. Consequently, we assert that, under these test conditions, a stress amplitude of 1350 MPa represents the fatigue strength of low-pressure treated samples fabricated from steel grades 1.3532, 1.5920, and 1.6587. Nevertheless, even the samples comprised of nickel-free steel 1.7243, following low-pressure treatment, i.e., without internal intergranular oxidation, exhibited superior fatigue properties compared to the much more expensive steel 1.3532, which contains a higher nickel content after classic gas carburizing.

Based on the presented results, it was difficult to establish a simple correlation between the amount of retained austenite and fatigue strength because steel 1.7243, containing the least amount of this austenite, showed a much lower core hardness than others after hardening, and the differences in fatigue test results for other tested grades were statistically insignificant. However, it seems that the presence of retained austenite is beneficial because, as a result of cyclic stresses, it is transformed into martensite, which was confirmed by the results of transmission electron microscopy examinations. These results are consistent with the results presented in [27], where carburized steel with a composition very similar to 1.6587 steel was tested. As a consequence of the mechanically induced phase transformation from austenite to martensite, it is evident that the austenite will eventually exhaust, and the mechanical energy will no longer be dissipated in this transformation. Consequently, the fatigue limit would exhibit a reduced value [28]. In order to test this effect, it would be appropriate to subject the layers tested here to tests following the Very High Cycle Fatigue protocol [29], potentially reaching up to 10^10^ cycles.

No significant differences were observed in the structure or fatigue properties of samples processed through the low-pressure LPC and LPCN methods. This lack of distinction could possibly be attributed to these differences being overshadowed by other factors or due to the relatively high uncertainties associated with the research methods, especially fatigue testing, employed.

It could also be mentioned that changing the processing technology from gas carburizing, which causes the IGO phenomenon, to vacuum carburizing brings greater benefits for fatigue properties than increasing the nickel content in steel.

## 5. Conclusions

- The chemical composition of the steel had a significant impact on both the structure of the layers and the properties of the carburized layers. The steel with the highest nickel concentration (1.3532) was carburized to the smallest depth and had the lowest surface concentration of carbon;
- The chemical composition also influenced the retained austenite content in the layers. The steel grade 1.3532, with the highest nickel content, exhibited a volumetric fraction of retained austenite exceeding 20%, whereas for the nickel-free steel grade (1.7243), it was only approximately 10%;
- Changing the processing method from gas carburizing, which induces the internal intergranular oxidation (IGO) phenomenon, to vacuum carburizing presents greater advantages for the fatigue properties than increasing the nickel content in the steel. Even samples made of nickel-free steel 1.7243 demonstrated, after low-pressure treatment, superior fatigue properties in comparison to conventional gas carburizing of the notably more expensive steel 1.3532, which had a higher nickel content;
- Under the specific test conditions applied, a stress amplitude of 1350 MPa represents the fatigue strength of low-pressure treated samples manufactured from nickel-containing steel grades, namely 1.3532, 1.5920, and 1.6587;
- Establishing a correlation between the retained austenite quantity and the fatigue strength was not straightforward. Nevertheless, the presence of retained austenite may be advantageous because it was shown that cyclic stress induced its transformation into martensite, a phenomenon confirmed by transmission electron microscopy examinations.

## Figures and Tables

**Table 1:** Chemical composition of steel grades used in this experiment measured with GDOES. Wt. %.

EN Steel Grade	C	Mn	Si	P	S	Cr	Ni	Mo	V	W	Cu	Al	Ti	Co	Nb
16NiCrMo16-5 (1.3532)	0.15	0.25	0.18	0.01	0.01	1.12	3.78	0.21	0.01	0.03	0.17	0.02	0.01	0.05	0.01
18CrNi8 (1.5920)	0.19	0.58	0.25	0.01	0.02	2.05	2.47	0.05	0.01	0.03	0.04	0.02	0.01	0.03	0.05
18CrNiMo7-6 (1.6587)	0.17	0.59	0.27	0.01	0.01	1.70	1.77	0.25	0.01	0.03	0.24	0.02	0.01	0.03	0.01
18CrMo4 (1.7243)	0.21	1.05	0.23	0.02	0.01	1.09	0.11	0.21	0.01	0.03	0.21	0.03	0.01	0.03	0.00

**Table 2:** Grain size numbers and Vickers hardness of steel grades in as-received condition used in this experiment.

Steel Grade	ASTM Grain Size No	HV30	Std. Dev.
1.3532	7.5	248	2
1.5920	7.5	158	3
1.6587	7	251	7
1.7243	8	286	1

**Table 3:** ASTM E112 [22] grain size number of the core.

Steel Grade	LPC	LPCN
1.3532	9	9
1.5920	9	9
1.6587	9.5	9.5
1.7243	9	9

**Table 4:** Retained austenite content calculated from XRD, [Vol. %].

Steel Grade	LPC	LPCN
1.3532	20	21
1.5920	19	20
1.6587	14	15
1.7243	09	10

**Table 5:** Superficial Vickers hardness for layers from low-pressure processes.

	LPC		LPCN	
Steel Grade	HV30	Std. Dev.	HV30	Std. Dev.
1.3532	606	7	622	12
1.5920	685	9	684	10
1.6587	676	10	670	15
1.7243	693	20	702	17

**Table 6:** Case hardness depth (CHD) and relative case depth (CHD/D). D—sample diameter = 12 mm.

Low-Pressure Heat Treatment	LPC		LPCN	
Steel Grade	CHD [mm]	CHD/D	CHD [mm]	CHD/D
1.3532	0.8 ± 0.1	0.067 ± 0.008	0.8 ± 0.1	0.067 ± 0.008
1.5920	1.1 ± 0.1	0.092 ± 0.008	1.1 ± 0.1	0.092 ± 0.008
1.6587	1.1 ± 0.1	0.092 ± 0.008	1.1 ± 0.1	0.092 ± 0.008
1.7243	1.1 ± 0.1	0.092 ± 0.008	1.1 ± 0.1	0.09 ± 0.008

**Table 7:** Calculated interplanar distances d and corresponding phase components.

Ring No	d Spacing [nm]	Phase Component
1	0.233	ω
2	0.203	α—b.c.c. Fe (110)
3	0.184	γ—f.c.c. Fe (200)
4	0.176	ω
5	0.148	α—b.c.c. Fe (200)
6	0.142	ω
7	0.133	γ—f.c.c. Fe (220)
8	0.116	α—b.c.c. Fe (211)
9	0.100	α—b.c.c. Fe (220)
10	0.090	α—b.c.c. Fe (310)

## Data Availability

Data are contained within the article.
